# YAP Ultralate Laser-Evoked Responses in Fibromyalgia: A Pilot Study in Patients with Small Fiber Pathology

**DOI:** 10.3390/jcm13113078

**Published:** 2024-05-24

**Authors:** Elena Ammendola, Silvia Giovanna Quitadamo, Emmanuella Ladisa, Giusy Tancredi, Adelchi Silvestri, Raffaella Lombardi, Giuseppe Lauria, Marina de Tommaso

**Affiliations:** 1 Neurophysiopathology Unit, DiBrain Department, Aldo Moro University, 70124 Bari, Italy; elena.ammendola@uniba.it (E.A.); sg.quitadamo@gmail.com (S.G.Q.); emmanuellemmanuellaladisa@gmail.com (E.L.); tancredi.giusy96@gmail.com (G.T.); silvestriadelchi93@gmail.com (A.S.); 2 Neuroalgology Unit, Fondazione IRCCS Istituto Neurologico Carlo Besta, 20133 Milan, Italy; raffaella.lombardi@istituto-besta.it (R.L.); giuseppe.lauriapinter@istituto-besta.it (G.L.); 3 Department of Medical Biotechnology and Translational Medicine, University of Milan, 20133 Milan, Italy

**Keywords:** fibromyalgia, laser-evoked potentials, skin biopsy, small fiber neuropathy

## Abstract

**Background**: The investigation of C-fiber-evoked ultralow-level responses (ULEPs) at somatic sites is difficult in clinical practice but may be useful in patients with small fiber neuropathy. Aim: The aim of the study was to investigate changes in LEPs and ULEPs in patients with fibromyalgia affected or not by abnormal intraepidermal innervation. **Methods**: We recorded LEPs and ULEPs of the hand, thigh and foot in 13 FM patients with a normal skin biopsy (NFM), 13 patients with a reduced intraepidermal nerve fiber density (IENFD) (AFM) and 13 age-matched controls. We used a YAP laser, changing the energy and spot size at the pain threshold for LEPs and at the heat threshold for ULEPs. **Results**: ULEPs occurred at a small number of sites in both the NFM and AFM groups compared to control subjects. The absence of ULEPs during foot stimulation was characteristic of AFM patients. The amplitude of LEPs and ULEPs was reduced in patients with AFM at the three stimulation sites, and a slight reduction was also observed in the NFM group. **Conclusions**: The present preliminary results confirmed the reliability of LEPs in detecting small fiber impairments. The complete absence of ULEPs in the upper and lower limbs, including the distal areas, could confirm the results of LEPs in patients with small fiber impairments. Further prospective studies in larger case series could confirm the present findings on the sensitivity of LEP amplitude and ULEP imaging in detecting small fiber impairments and the development of IENFD in FM patients.

## 1. Introduction

An examination of the nociceptive pathways is of crucial importance in patients with chronic pain. Laser-evoked potentials (LEPs) are a reliable tool to analyze the involvement of Aδ fibers in neuropathic pain and small fiber pathology [1], using a skin biopsy to confirm the reduction in the intraepidermal nerve fiber density (IENFD) [2]. However, the neurophysiological assessment of C-fibers is quite difficult, as the slow conduction velocity and low amplitude of evoked responses may reduce the reliability of ULEPs outside the facial area [3]. Moreover, the coactivation of Aδ fibers masks the slower C-related response, as the general principle of cortical functioning is “first come, first served”. However, the use of stimulation methods capable of selectively activating C receptors could allow the detection of a late cortical response that is generally distinct from the Aδ potential [4]. In a recent article, we used a YAP laser with a thermal, non-painful intensity and a large spot, and observed an ultralate cortical response on the hand and leg in the majority of healthy volunteers [4]. Solid-state laser radiation has deeper penetration within the dermis, reducing superficial burns, with an advantage for clinical use [5].

The impairment of the small afferent fibers is often associated with fibromyalgia (FM) [6]. Recently, experienced neurologists in the field of pain have proposed the diagnostic evaluation of small fiber involvement in FM [1]. To determine the presence of small fiber impairment, it is recommended to use at least two of the following methods: heart rate variability, sympathetic skin response, Aδ-related evoked responses (LEPs), corneal confocal microscopy and skin biopsy [1].

The results of the skin biopsy are primarily based on the involvement of C-fibers. The use of laser-evoked potentials was recommended in patients with small fiber impairment [5], while the same authors stated that a single method does not completely describe the status of small afferents, so a more specific assessment of C fibers together with A delta fibers could be useful in patients with FM.

Neurophysiological studies with microneurography assessed the dysfunction of C fibers in patients with FM, which correlated with the severity of the disease [7,8]. Also, quantitative sensory testing confirmed the abnormal C-related thermal and painful sensibilities [6]. Based on previous observations, we considered conducting a pilot study to assess the function of Aδ and C fibers with the Nd:YAP laser in subgroups of fibromyalgia patients with varying degrees of cutaneous innervation in skin biopsy compared to a group of healthy subjects. The aim was to find a reliable neurophysiological signature of small fiber impairment in fibromyalgia patients.

## 2. Materials and Methods

### 2.1. Subjects

Twenty-six participants among those who had received a diagnosis of FM according to the 2016 criteria [9] agreed to undergo standard LEP and skin biopsy examination with recording of ULEPs. Two subgroups of patients were considered: fibromyalgia patients a with normal skin biopsy (FMN) and fibromyalgia patients with an altered biopsy (FMA). In addition, 13 age-matched healthy volunteers (4 men and 13 women) were included as a control group.

The study was conducted according to the rules of the Declaration of Helsinki of 1975 (https://www.wma.net/what-we-do/medical-ethics/declaration-of-helsinki/), revised in 2013. The study on skin biopsies and laser-evoked potentials was originally approved by the ethics committee of the General Hospital of Bari Policlinico in 2012. The continuation of the study was approved on December 2022. All study participants gave their informed consent, specifically reporting the possible occurrence of transitory superficial skin lesions.

### 2.2. Skin Biopsy

The laboratory procedure we used is described in the work of Devigili et al. [2] and in our earlier studies [10,11] (Appendix A).

The fibromyalgia patients were divided into two groups based on the results of the skin biopsy performed at two sites on the proximal and distal thigh: normal pattern (N = normal) and neuropathic pattern (A = abnormal). The groups proved to be homogeneous; each group consisted of 13 people. In the AFM group, 12 patients had non-length-dependent neuropathy and 1 patient had length-dependent neuropathy.

### 2.3. Laser Stimulation

For this study, we used the STIMUL 1340 system from Electronic Engineering^®^ (El.En. S.p.A., Florence, Italy). It was equipped with two laser sources: a neodymium:yttrium aluminum perovskite laser (Nd:YAP), which emits an infrared beam, and a coaxial diode laser source, which emits a visible red beam. The stimulation procedure was similar to a previous study performed in our laboratory [4]. In this case, only the right side was stimulated to increase compliance for this type of procedure, which is known for its relatively long duration (approximately 100 min in total) and discomfort.

The parameters were set to activate the small myelinated afferents (Aδ) or the non-myelinated afferents (C).

The details of the stimulation method are described in Appendix B.

### 2.4. Recording

The recording methods were already described [4]. In brief, the EEG was recorded with 62 Ag–AgCl electrodes placed on the scalp with a prewired cap, according to the international 10–20 system. One electrode on the bridge of the nose served as a reference. The ground electrode was placed on the right forearm. Two electrodes placed on the upper left and lower right side of both eyes monitored eye movements and blinking. The impedance was kept below 5 kΩ.

### 2.5. LEP Analysis

To process the LEP data, we used the MATLAB platform and the toolboxes EEGLAB 14_1_1 and LetsWave version 7. First, we performed epoch splitting and baseline correction in the time domain (−0.1–2 s). An automatic exclusion method was set for eye movements based on EOG channels and for signals exceeding 100 µV. We applied the filters with a bandpass at 1 Hz–30 Hz; a notch filter removed the power line noise artifacts at 50 Hz (low: 48, high: 52). We visually inspected and removed bad channels and then interpolated with the neighboring electrodes. At this point, we evaluated the average of at least 21 artifact-free trials for individual responses at each stimulation site.

The latencies of LEPs N2–P2 and the ULEPs positivity wave were measured considering the maximum peak on the Cz channel.

We visually identified the main peaks in the C-related potentials. Not all patients showed C-related potentials. In cases in which an average positive response occurred at the vertex that clearly stood out from the signal noise, we determined the maximum positivity (ULEP P) considering the interval 700–2000 msec. In the present study, we did not consider those potentials that could be caused by the coactivation of Aδ fibers [4]. Therefore, the latencies of the ULEP P were measured at the maximum peak in the given interval for each subject on the Cz channel.

The amplitudes of LEPs N2–P2 and the ULEP positivity wave were evaluated on the 62 channels, considering the maximum peaks at the given intervals.

### 2.6. Clinical Evaluation

We tested the patients with the following scales: visual analogue scale (VAS) [12], Zung SDS and SAS [13,14], fibromyalgia-induced disability questionnaire (FIQ) [15], multidimensional assessment of fatigue (MAF) [16] and brief pain inventory (BPI) with subscales for pain severity and interference [17].

#### Statistical Procedure

The latencies of the Aδ-related N2–P2 complex and C-related ultralate positivity, as well as pain and heat thresholds were compared between the 3 groups using one-sided ANOVA with a post hoc Bonferroni test.

In addition, a chi-square test for independence between groups was performed to test for the presence of LEPs and ULEPs at the different stimulation sites.

The amplitude of the C-related ultralate potential and the Aδ-related components of N2 and P2 were compared between groups using the statistical package in the LetsWave vers. 7 tool. For the comparison between the controls and the AFM and FMN groups, the unpaired *t*-test with cluster-based permutation was applied at a threshold of 0.05 and 2000 permutations.

## 3. Results

We analyzed LEPs and ULEPs from 26 FM patients, 13 with a normal IENFD (NFM) (2 males aged 46.54 + 16.4), 13 with an abnormal skin biopsy (AFM) (2 males aged 54.62 + 9.87) and 13 age-matched controls (4 males; age 46.54 + 9.6. ANOVA F 2.47 *p* 0.16). In all cases, local skin burning resolved within 15 days.

We noted the pain and heat thresholds and the presence of vertex reactions of a-delta modality of the stimulation. The pain threshold for the stimulation of the Aδ fibers was similar in both groups. Perceived pain was increased in both FM groups compared to controls when the knee was stimulated (Table 1; Supplementary Table S1).

**Figure 1:**
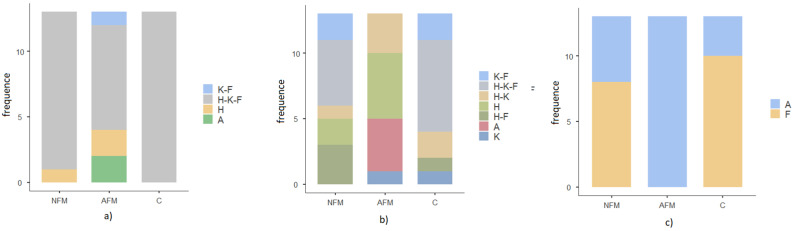
Presence of laser-evoked responses (LEPs) of a-delta fibers and laser-evoked responses (ULEPs) of C-fibers in 13 healthy controls (C), 13 fibromyalgia patients with normal skin biopsy (NFM) and 13 fibromyalgia patients with abnormal skin biopsy (AFM) for stimulation of the hand (H), knee (K) and foot (F). In (**a**), we showed the absence of any detectable waves and in (**b**) the presence of ULEPs. In (**c**), we showed the presence of ULEPs for foot stimulation. No patient with AFM had ULEPs during foot stimulation.

All healthy subjects showed responses after the stimulation of the Aδ-fibers at the three stimulated sites; one patient with a normal skin biopsy had no LEPs at two stimulation sites (7.6%), 4 FM patients with abnormal skin biopsies had no LEPs at least at one site (two at two sites and two at all sites, 30.7%); (Chi-square test 9.27 *p* 0.15); (Supplementary Table S1, Figure 1a).

In FM patients, the absence of LEPs was consistent with the absence of ULEPs in the same stimulation region.

### 3.1. C-Modality of Stimulation

In the C modality of stimulation, the heat threshold was similar between the knee and foot stimulation groups, while the NFM group had a lower heat threshold. However, the Bonferroni test was not significant (Table 2; Supplementary Table S2).

In almost all cases, the late positivity was preceded by an early negative–positive complex. This was probably generated by the coactivation of Aδ fibers, as we had previously observed in healthy controls (see Ammendola et al., 2023) [4]. In the present study, we considered the late response attributable to C-fiber activation. In patients with a normal skin biopsy, the presence of ULEPs was 84.6% in the hand, 61.5% in the knee and 76.9% in the foot. Only five patients had ULEPs in the three stimulated sites; six patients had the ultralate potential in two sites: one in the hand and knee, two in the knee and foot and three in the hand and foot; two patients had ULEPs in the hand only (Supplementary Table S2, Figure 1b).

In the patients with an abnormal skin biopsy, the presence of ULEPs was 61.5% on the hand and 30.7% on the knee. Three patients had ULEPs on the hand and knee together, five on the hand only and one on the knee only; four had reactions at none of the stimulation sites (Figure 1b). The presence of ULEPs was different in the three groups (chi-square 27.4 *p* 0.007; Figure 1b). None of the thirteen patients with an abnormal skin biopsy had ULEPs at the foot level (chi-square 17.3 *p* < 0.0001; Figure 1c).

For the amplitude of the parietal waves, at the hand level, we found a slight reduction in the Aδ-related N2 amplitude in FMN patients compared to controls, which was restricted to the central regions, whereas AFM patients showed a reduced N2 and P2 amplitude over fronto-central and parietal electrodes.

**Figure 2:**
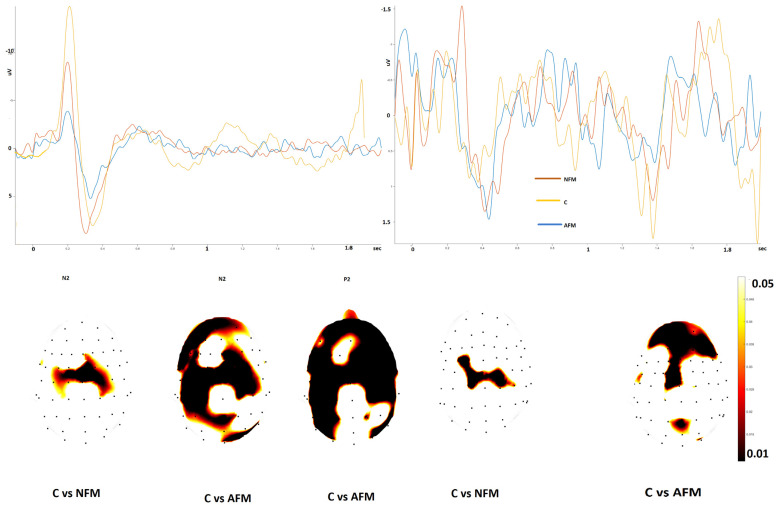
Left side: grand average of the N2–P2 complex from the hand in fibromyalgia with normal skin biopsy (NFM), abnormal skin biopsy (AFM) and controls (C). The lower part of the figure shows the statistical probability maps expressing the *t*-test. Right side: grand average of the ultralate responses obtained by hand in the three groups. The statistical probability maps express the results of the *t*-test applied to the ultralate positivity (*t*-test with cluster-based permutation was applied at a threshold of 0.05 and 2000 permutations).

The amplitude of the ULEPs was also reduced in the FMN patients, in a region restricted to the vertex and in the parietal and frontal regions of the AFM group (Figure 2).

**Figure 3:**
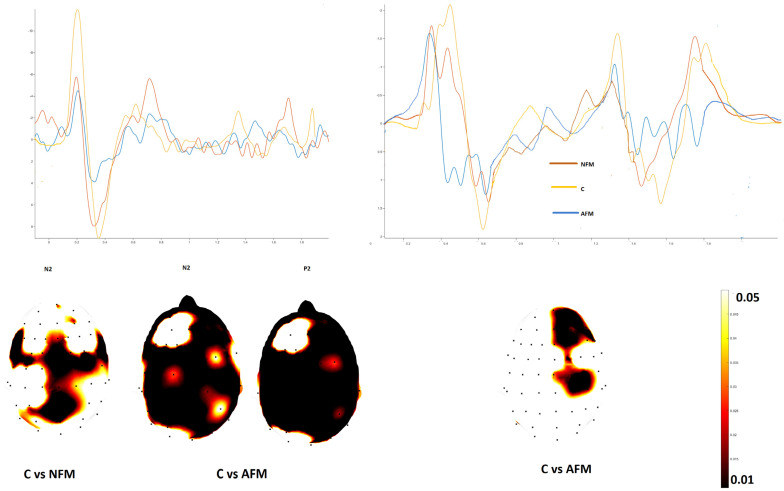
Left side: grand average of the N2–P2 complex from the thigh in fibromyalgia with normal skin biopsy (NFM), abnormal skin biopsy (AFM) and controls (C). Statistical probability maps expressing the *t*-test for the N2 and P2 components are shown in the lower part of the figure. Right side: grand average of the ultralate responses obtained by hand in the three groups. The statistical probability maps express the results of the *t*-test applied to the ultralate positivity (*t*-test with cluster-based permutation was applied at a threshold of 0.05 and 2000 permutations).

The knee stimulation in the Aδ modality elicited an N2 wave with a reduced amplitude in the centro-parietal regions in FMN patients and a diffuse reduction in N2 and P2 waves in AFM patients compared to controls (Figure 3).

ULEPs appeared reduced in the AMF group compared to controls over the centro-frontal leads (Figure 3), whereas there was no significant difference in amplitude in the FMN group.

**Figure 4:**
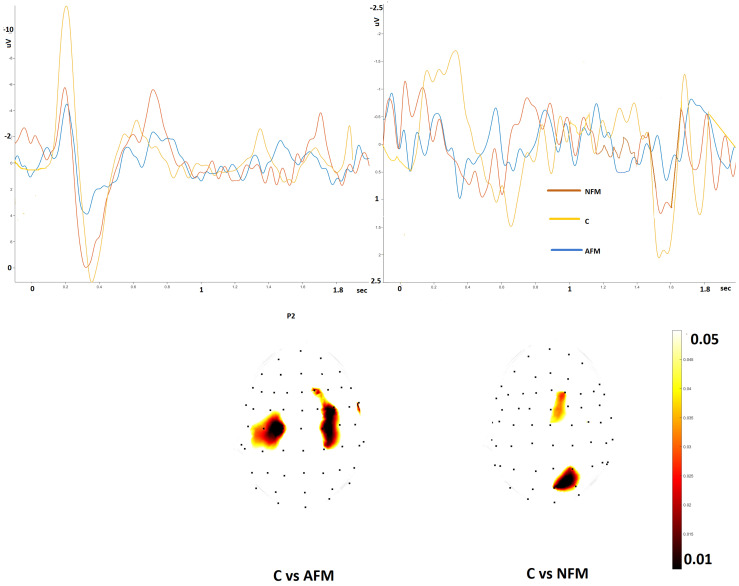
Left side: grand average of the N2–P2 complex from the foot in fibromyalgia with normal skin biopsy (NFM), abnormal skin biopsy (AFM) and controls (C). Statistical probability maps expressing the *t*-test for the P2 component are shown in the lower part of the figure. Right side: grand average of the ultralate responses obtained by hand in the three groups. The statistical probability maps express the results of the *t*-test applied to the ultralate positivity (*t*-test with cluster-based permutation was applied at a threshold of 0.05 and 2000 permutations).

The foot stimulation in the Aδ modality resulted in a reduced P2 amplitude in the bilateral central leads in the AFM patients (Figure 4).

The ULEPs were absent in the AFM group and reduced in a limited zone within the central and parietal regions in the FMN group (Figure 4).

The latencies of LEPs and ULEPs, if present, were similar in the groups (Supplementary Table S3).

### 3.2. Correlation with Clinical Characteristics

The MANOVA analysis comparing the main clinical characteristics between the FM groups approached statistical significance (F 2.95 *p* 0.056). Notably, disease history was longer in the AFM group (patients with reduced IENFD had higher WPI scores (AFM 14 + 3.2; NFM 8.2 + 2.3 years F 4.79 *p* 0.01)) (Supplementary Table S3).

We found a positive correlation between the amplitude of knee-evoked ULEPs and the MAF score for fatigue (Pearson—0.71 *p* 0.05; linear regression test R2 2.14 t 2.81 *p* 0.018). There was no relevant correlation between the IENFD values and the latencies and amplitudes of the LEPs and ULEPs.

## 4. Discussion

The present study followed on from a recent observation by our group on ultralate potentials elicited with the YAP laser at low intensity and an enlarged spot in normal controls [4]. In this study, we observed late responses in the trigeminal, upper and lower extremities in a congruent number of healthy subjects. Although they were preceded by an early positive–negative complex, indicating the probable coactivation of a-delta fibers during single sets of stimulation, their latency was consistent with the activation of C-fibers. Now, we wanted to test the same stimulation paradigm in patients with small fiber involvement confirmed through a recent skin biopsy. To this end, we studied patients with a clinical diagnosis of fibromyalgia who were divided into two subgroups, one with a normal and the other with a reduced IENFD in at least one site of the lower extremities, and an age-matched control group. We found that ULEPs were less common in FM patients than in control subjects, and that this was particularly evident in patients with abnormal IENFD values. At the sites where they were detectable, ULEPs were reduced in amplitude in both FM groups, but this tendency predominated in patients with abnormal skin biopsies. The reduction in the amplitude of ULEPs detected when the thigh was stimulated corresponded to a higher degree of fatigue in the entire FM sample. The following sections explained the results in detail.

### 4.1. Pain and Heat Threshold

Pain threshold was similar in both FM groups compared to controls, but pain perception was increased in both groups compared to controls on the thigh corresponding to the knee trigger point. This finding reproduced what has been described in previous studies [11,18], namely, a normal Aδ-fiber pinprick threshold in FM patients, with an enhancement in pain perception at critical points.

The heat threshold appeared to be slightly increased in FM patients with small fiber involvement, particularly at the hand and knee. However, this did not reach statistical significance, as the number of subjects was small and the subjective sensation of FM patients rarely corresponded to a neuropathic profile, a phenomenon that has been described in previous studies [18].

### 4.2. Presence of LEPs and ULEPs in FM Patients

While most FM patients, regardless of IENFD, had measurable LEPs on the hand and lower limbs, ULEPs were undetectable in a consistent group of FM patients with an abnormal skin biopsy, and even patients with a normal IENFD had a lower number of sites where ULEPs could be reliably detected compared to controls. According to previous studies on skin biopsies in FM patients [11,19,20], the predominant site of denervation was the proximal part of the leg. However, no patient with an abnormal skin biopsy showed ULEPs from the foot stimulation, probably because the generation of detectable ULEPs from the distal part of the leg might have required a constant number of activated C-fibers to bypass signal scattering along the afferent pathway. In this sense, a subtle C-fiber dysfunction in FM patients could also exist at the distal site of stimulation. ULEPs from the proximal leg stimulation site were present in a small number of patients with proximal small fiber denervation, albeit with a reduced amplitude, as we commented below. The complete absence of ULEPs at the somatic stimulation sites of the upper and lower limbs could be a sign of denervation of the small fibers in patients with FM, a statement that should be further confirmed in larger patient series.

### 4.3. LEP and ULEP Latencies and Amplitudes

In agreement with previous studies, we found no latency abnormalities of the cortical complex associated with the vertex Aδ and C in FM patients [11,19,20], as demyelination along the spino-thalamic tract is not typical of FM. In contrast to our recent study, we found a decrease in Aδ N2. In agreement with previous studies, we found no latency abnormalities of the cortical complex associated with vertex Aδ and C in FM patients [11,19,20], as demyelination along the spino-thalamic tract is not typical of FM. In contrast to our recent study, we found a decrease in the Aδ N2 amplitude upon the stimulation of the thigh and hand in FM patients with a normal skin biopsy [21], while the P2 amplitude was within normal values. In the present study, we used a statistical analysis with multiple channels, which could highlight the amplitude differences compared to a single-channel analysis. A visual inspection of the overall average revealed a reduced N2–P2 complex in NFM patients, but as discussed in previous studies [4,11], there is a large variability in FM patients for the phenomenon of reduced habituation with repeated stimulation. In this study, we did not calculate habituation across individual trials because we included ULEPs, which are rarely recognized as single responses [4]. In patients with an IENFD reduction, the amplitude of ULEPs was reduced at all stimulation sites compared to controls, indicating a dysfunction of Aδ fibers. The amplitude of ULEPs, when present, was reduced in patients with small fiber involvement, but also in patients with a normal IENFD, indicating a likely subtle and initial dysfunction of small fibers that could develop over time. Indeed, disease duration was shorter in patients with a normal skin biopsy than in patients with small fiber involvement.

### 4.4. Correlation with Clinical Features

In agreement with previous studies [11,18], involvement of the small fibers did not correspond to a more severe FM phenotype. FM is a complex syndrome which does not resemble a model of neuropathic pain but is rather an example of nociplastic pain in which phenomena of central sensitization may predominate over the purely neuropathic aspects of the disease. The correlation we found between the decrease in ULEP amplitude and fatigue confirmed previous findings of a possible influence of small fiber dysfunction on motor performance [22,23], even considering the limitation due to the small number of patients (see comments below).

### 4.5. Limitation of the Study

This was a pilot study to investigate the reliability of C-related ULEPs in patients with proven small fiber impairment. The number of patients was very small, as was the number of age-matched controls. Some comparisons approaching statistical significance could find confirmation in larger case series. We did not include the early N1 component as we wanted to focus on the main vertex components in the analysis. In agreement with previous findings [4], we observed an early vertex complex in patients and controls consistent with the coactivation of Aδ fibers also in the C modality of the stimulation, which was not included in the analysis due to its unclear origin. However, the presence of this early complex could reduce the reliability of somatic C-related ULEPs.

## 5. Conclusions

The present preliminary results may confirm the reliability of LEPs in detecting small fiber impairment, as the Aδ-related vertex complex amplitude reduction characterizes patients with small fiber impairment and likely predicts small fiber involvement during FM development. Although the signal-to-noise ratio of ULEPs elicited from somatic sites was low, they were detectable at a consistent number of stimulation sites in control subjects, and their complete absence in the upper and lower limbs, including distal sites, may underpin the findings of LEPs in patients with small fiber impairment.

Further prospective studies in larger case series could confirm the present findings on the sensitivity of LEP amplitude and ULEP imaging in detecting small fiber impairment and the development of IENFD in FM patients.

## Figures and Tables

**Table 1:** Mean, SD and SEM of pain threshold (T) expressed in Joule (J) and pain sensation with 0–10 VAS for the sites of stimulation in Aδ fiber modality. NFM: fibromyalgia patients with normal skin biopsy. AFM: fibromyalgia patients with abnormal skin biopsy. C: controls.

Site of Stimulation	Type of Subjects	N	Mean	SD	SE	ANOVA
HAND T (J)	NFM	13	6.31	1.97	0.548	F 1.63 *p* 0.22
	AFM	13	6.77	3.79	1.051	
	C	13	8.54	3.93	1.090	
HAND VAS	NFM	13	74.38	15.63	4.335	F 1.88 *p* 0.17
	AFM	13	79.54	14.88	4.126	
	C	13	66.46	18.81	5.218	
KNEE T (J)	NFM	13	3.54	1.98	0.550	F 1.99 *p* 0.15
	AFM	13	5.31	2.84	0.788	
	C	13	3.54	1.85	0.514	
KNEE VAS	NFM	13	81.00	12.14	3.367	**F 4.89** *p* 0.017 *
	AFM	13	82.54	10.85	3.010	
	C	13	64.38	18.25	5.062	
FOOT T (J)	NFM	13	6.85	2.76	0.767	F 0.277 *p* 0.76
	AFM	13	7.77	4.00	1.110	
	C	13	7.54	3.84	1.066	
FOOT VAS	NFM	13	76.62	13.29	3.686	F 0.777 *p* 0.47
	AFM	13	78.69	11.10	3.079	
	C	13	73.46	10.20	2.830	

* Post hoc Bonferroni test NFM vs. C *p* 0.013; AFM vs. C *p* 0.006.

**Table 2:** Warm threshold (T) expressed in Joule (J) for the three sites of stimulation in the C-fiber modality.

Site of Stimulation	Type of Subjects	N	Mean	SD	SE	ANOVA
HAND T (J)	NFM	13	6.62	3.43	0.951	**F 3.63** *p* 0.046 *
	AFM	13	8.08	3.35	0.930	
	C	13	5.92	6.10	1.693	
KNEE T (J)	NFM	13	5.77	3.79	1.051	F 3.25 *p* 0.058
	AFM	13	9.62	4.25	1.180	
	C	13	6.00	5.64	1.565	
FOOT T (J)	NFM	13	11.00	2.35	0.650	F *p* 0.89 *p* 0.42
	AFM	13	12.46	3.23	0.896	
	C	13	12.00	5.07	1.405	

* Bonferroni test not significant. NFM—FM patients with normal skin biopsy AFM—patients with reduced intraepidermal nerve fiber density; C—controls.

## Data Availability

Neurophysiological, skin biopsy and clinical data are available on request to corresponding author.

## Supplementary Materials

The following supporting information can be downloaded at: https://www.mdpi.com/article/10.3390/jcm13113078/s1, Supplementary Methods, Table S1: Pain thresholds for a-delta fibers modality of stimulation in FM patients (J: joules, H: hand, K: knee and F: foot); Table S2: Stimulation parameters of C-LEPs (J: joules, H: hand, K: knee and F: foot); Table S3: Neurophysiological and clinical features in AFM and NFM patients. BPI Brain Pain Inventory. MAF; Multidimensional Assessment of Fatigue; NRS: Numerical Rating Scale; WPI Wide Pain Index IENFD Intraepidermal Nerve Fober Density.

## Appendix A. Skin Biopsy

The skin biopsy involved the removal of a 3 mm skin flap from the thigh 20 cm below the anterior iliac spine and in the distal third of the leg (10 cm above the lateral malleolus in the area of the sural nerve). The procedure was performed under local anesthesia with an intradermal injection of lidocaine and required no sutures. The biopsy samples were processed to obtain a quantification of the small nerve fibers. The laboratory procedure described in the work of Devigili et al. [2] included a sample preparation phase: fixation with 2% paraformaldehyde lysine periodate at 4 °C overnight, cryoprotection, sectioning and finally immunostaining with the 9.5 anti-polyclonal protein product. The counting of small nerve fibers was performed with a semi-automatic counter on three non-consecutive central sections under a bright-field optical microscope [2]. The data obtained on the number of small nerve fibers were compared with normative values determined in adult subjects on the basis of gender and age groups [10].

## Appendix B. Laser Stimulation

For this study, we used the STIMUL 1340 system from Electronic Engineering^®^ (El.En. S.p.A., Florence, Italy). It was equipped with two laser sources: a neodymium:yttriumaluminum perovskite laser (Nd:YAP), which emits an infrared beam, and a coaxial diode laser source, which emits a visible red beam. The system works with a fiber optic conductor. We performed the recordings with a high-resolution EEG headset equipped with 62 recording electrodes (Micromed Brain Quick device, Micromed S.p.A., Treviso, Italy). The laser stimuli were administered at three sites on the right side of the body: on the dorsum of the hand, on the dorsum of the foot and on the proximal anterolateral segment of the lower limb near the knee. The stimulation procedure was similar to a previous study conducted in our laboratory [4]. In this case, only the right side was stimulated to increase compliance for this type of procedure, which is known for its relatively long duration (approximately 100 min in total) and discomfort, especially in individuals with altered pain perception, such as those with fibromyalgia.

The parameters were set to activate the small myelinated afferents (Aδ) or the unmyelinated afferents (C).

In brief, for each stimulated area and for each of the two modalities under study (Aδ and C), at least 25 consecutive stimulations were delivered with an interstimulus interval (ISI) of at least 10 s. To avoid the phenomenon of habituation and to minimize the skin damage caused by the laser beam, the laser beam was skillfully guided to nearby and never identical skin areas.

The Nd:YAP stimulator could elicit the stimulation of both types of small nerve fibers by modulating certain stimulation parameters: a higher stimulation intensity (10.19–26.74 J/cm^2^), shorter duration (5 ms) and a small irradiated area (diameter 5 mm) to elicit the pinprick sensation; a lower intensity (7.46–13.37 J/cm^2^), longer duration (10 ms) and a larger skin area (diameter 10 mm) to elicit only the heat sensation. The diameter of the illuminated area was measured with an infrared-sensitive paper and was 5 mm for Aδ-LEPs and 10 mm for C-LEPs. The threshold for delivered energy was individually adjusted and increased by 0.25 J per step until the subject clearly felt a pure warm sensation for C-fibers, whereas for the Aδ stimulation, after the initial sensation of a general feeling of pain, we delivered energy until the subjects clearly felt the corresponding stinging sensation on the pain rating scale with values between three and six.

Each subject was asked to pay attention to the two different types of sensations elicited by the stimulator: pinprick pain for the Aδ modality and pure heat sensations for the C modality. During the stimulation of the C fibers, the patient was instructed to alert the neurophysiology technician if they perceived any sensation other than warmth that may occur during the involuntary co-stimulation of the Aδ fibers, such as a stabbing or burning pain. Although we only had the skin biopsy results from the lower limb, we decided to test the upper limb with the YAP laser as well as the extremity, as the IENFD reduction could also affect this area [8]. The stimulation paradigm provided for randomization between sites and the modality of stimulation.
