# Supplementary material for: YAP Ultralate Laser-Evoked Responses in Fibromyalgia: A Pilot Study in Patients with Small Fiber Pathology
Source: J Clin Med. 2024 May 24;13(11):3078. doi: 10.3390/jcm13113078 (PMC11173050; doi:10.3390/jcm13113078)
Supplement: Supplementary file 1 [file jcm-13-03078-s001.zip › jcm-2961430-supplementary.pdf]

Table S1. Pain thresholds for a-delta fibers modality of stimulation in FM patients (J: joules, H: hand, K: knee and F: foot).

Non-neuropathic patients (FMN)	Gender	Age (years)	Hand (J)	VAS	Knee (J)	VAS	Foot (J)	VAS	Absence LEP in stimulation sites
1	F	78	3.5	68	3.5	76	3.75	89	
2	F	32	4.5	76	5.5	74	5	76	
3	F	42	2.75	85	2.5	88	3	90	
4	F	40	4.5	50	4	96	4.5	63	
5	F	21	4.25	84	3.75	92	4.25	88	
6	M	43	4	94	3.75	94	3.25	87	K-F
7	F	56	2.5	100	3.75	80	3	79	
8	F	40	2.75	50	2.75	96	2.75	63	
9	F	78	3.5	71	3.5	88	3	77	
10	F	42	2.75	90	3	68	3.25	62	
11	F	47	3	65	3.5	59	3.75	71	
12	F	43	2.25	67	2.5	70	3.5	97	
13	F	43	3.5	67	3.25	72	3.25	54	
mean		46,5	3,5	74,4	3,5	81	3,5	76,6	
**Neuropathic patients (AFM)**	**Gender**	**Age (years)**	**Hand (J)**	**VAS**	**Knee (J)**	**VAS**	**Foot (J)**	**VAS**	**Absence LEP in stimulation sites**
14	F	50	4.75	89	4.25	83	4.75	93	
15	F	73	2	92	3.5	75	3	64	K-F
16	F	43	3.5	63	3.75	89	4.25	68	
17	F	55	5.25	82	5.5	85	5.25	82	
18	F	66	2.75	78	2.75	71	2.75	63	H-K-F
19	M	46	3	97	3.75	100	3.75	95	
20	F	41	3.25	64	3	71	4	76	
21	F	42	4.25	97	4.5	90	4	72	H-K-F
22	F	58	4.25	64	4.25	80	4	83	
23	F	64	3	98	3	61	3	85	
24	F	59	3.5	55	3	90	3.5	70	
25	M	56	2	84	2.25	95	2.25	78	K-F
26	F	57	2.5	71	2.75	83	3.25	94	
mean		54,6	3,4	79,5	3,6	82,3	3,7	78,7	
**Healthy controls (C)**	**Gender**	**Age (years)**	**Hand (J)**	**VAS**	**Knee (J)**	**VAS**	**Foot (J)**	**VAS**	**Absence LEP in stimulation sites**
1	F	64	2.25	28	2.75	90	2.75	57	
2	F	53	2.75	62	2.5	63	3	75	
3	M	51	2.5	75	3.75	75	4	75	
4	F	41	3.75	75	3.5	80	3.5	73	
5	F	49	3.25	87	3	80	2.5	90	
6	F	50	3.5	90	4	65	3.5	86	

7	F	31	2.75	69	3	48	3.5	70	
8	F	47	3.5	60	2.75	53	3	84	
9	M	43	3.75	64	3.5	60	3.75	70	
10	F	44	2.75	79	2.75	27	3.25	80	
11	M	41	3.25	70	3	55	4	55	
12	F	60	4	30	3.5	51	5	70	
13	M	31	3	75	4	90	3.75	70	

Table S2. Stimulation parameters of C-LEPs (J: joules, H: hand, K: knee and F: foot).

**Non-neuropathic patients (FMN)**	**Gender**	**Age (years)**	**Hand (J)**	**Knee (J)**	**Foot (J)**	**Presence U-LEP in stimulation sites**
1	F	78	6,5	7,5	8	H-K
2	F	32	8,25	10,25	10,25	H-K-F
3	F	42	7,25	7	7,25	H-K-F
4	F	40	6,5	7	7,5	K-F
5	F	21	6,5	6,5	8,5	K-F
6	M	43	6,5	7	7,75	H
7	F	56	7	7,5	7	H-K-F
8	F	40	7	7,5	9	H-K-F
9	F	78	10,5	10,25	10	H
10	F	42	7	7,25	7,75	H-K-F
11	F	47	9,5	10	10,5	H-F
12	F	43	8,75	8,75	10,5	H-F
13	F	43	7,25	7,25	7,75	H-F
mean		46,5	7	7,5	8,5	
**Neuropathic patients (AFM)**	**Gender**	**Age (years)**	**Hand (J)**	**Knee (J)**	**Foot (J)**	**Presence U-LEP in stimulation sites**
14	F	50	8	8,5	8,5	no U-LEPs
15	F	73	9,5	10,25	10,5	H
16	F	43	7	9	8	H
17	F	55	9,5	9,25	9,5	H
18	F	66	8,5	8	10	no U-LEPs
19	M	46	6,75	7,5	8,75	H
20	F	41	8,25	8,25	8	H
21	F	42	8,25	9,25	8,75	no U-LEPs
22	F	58	9,5	9,25	9,75	no U-LEPs
23	F	64	7	8	10	K
24	F	59	9,25	9	9,5	H-K
25	M	56	8,75	9	9	H-K
26	F	57	6	8,25	8,5	H-K
Mean		54,6	8,2	8,7	9,1	

Healthy controls (C)	Gender	Age (years)	Hand (J)	Knee (J)	Foot (J)	Presence U-LEP in stimulation sites
1	F	64	1,75	2	5	T-K-F
2	F	22	2,75	3,75	5	T-H-K-F
3	M	53	3	4,75	7	T-H-K-F
4	F	23	7,5	7	7,5	T-K-F
5	F	51	7,25	7,5	9,75	H-K-F
6	F	22	6,75	7,5	8,75	T-H-K-F
7	F	24	7	5,25	6,5	T-H-F
8	F	41	4,75	7	7,5	T-H-K-F
9	M	49	5,5	6,5	8,25	T-K
10	F	50	7,25	6	7,5	T-H-K-F
11	M	27	6,25	7	7,75	T-H-K-F
12	F	31	8,25	9,75	10	H-K-F
13	M	47	7,25	8,25	8	T-H-K
14	F	23	6,25	6,25	6,25	T-H-F
15	F	22	8	8,25	9,25	T-H-K-F
16	M	43	8,25	8,75	9,25	T-H-K-F
17	F	26	7,25	7	10,25	T-K-F
18	M	41	6,75	7,5	7	T-H-K-F
19	M	29	8,75	8,5	10,5	T-H-K
20	F	31	5,25	5,25	6,5	T-H-F
Mean		36	6,2875	6,6875	7,875	

	Age (years)	Hand AD-LEPs		Hand AD-LEPs		Hand C-ULEPs		Knee AD-LEPs		Knee AD-LEPs		Knee C-ULEPs		Foot AD-LEPs		Foot AD-LEPs		Foot C-ULEPs		Duration Illness (years)	BPI			MAF	FIQ	NRS	VPI	IENFD	
		N2		P2		P2		N2		P2		P2		N2		P2		P2			IDB	IVE	IAL					proximal	distal
		ms	uV	ms	uV	Ms	uV	ms	uV	ms	uV	ms	uV	ms	uV	ms	uV	ms	uV										
AFM	51	0.19	-15.9	0.28	18.5	1.6	2.07	0.2	-12.5	0.34	13.9			0.22	-14.9	0.34	8.47			20	30	12	21	32	43	8	19	10.5	7.6
	73	0.31	0.59	0.43	7.58			0.28	-3.89	0.46	2.98			0.24	-0.57	0.42	7.26			30	30	13	21	30	47	8	14	9.6	9.7
	66	0.35	4.02	0.42	8.13			0.2	-1.4	0.26	7.8			0.17	-4.02	0.32	6.44			5	32	20	26	6	45	6	13	11.9	7.8
	43	0.25	-9.87	0.33	11.3	1.4	2.88	0.22	-11.1	0.38	7.98			0.25	-5.16	0.36	11.1			7	30	20	20	41	63	10	12	12.5	12.3
	55	0.42	-1.85	0.49	4.26			0.35	-2.28	0.47	3.16			0.4	-4.15	0.59	4.73			12	37	24	24	37	68	8	16	11.2	6.6
	41	0.19	-12.5	0.28	16.7	1.8	3.7	0.19	-11.2	0.3	10.3			0.21	-12.9	0.33	13.4			15	37	19	22	30	61	8	12	8.2	7.2
	46	0.26	-5.33	0.42	7.86			0.24	-11.5	0.44	2.11			0.4	-2.09	0.47	1.96			7	37	24	30	50	94	10	18	11.9	7.8
	64																			15	35	24	24	46	82	9	11	12.2	7.1
	57	0.22	-6.4	0.32	5.51	0.86	7.07	0.24	-6.24	0.47	8.79	1,00	9.59	0.3	-2.55	0.56	13.7			5	33	6	13	31	65	9	17	6.7	7.7
	58	0.26	-3.88	0.36	13.3	1.3	1.87	0.24	-2.66	0.34	13.2			0.29	-0.61	0.44	9.82			29	32	19	22	47	73	10	16	9.3	13.5
	64	0.2	-3.03	0.32	17.8	1.4	3.66	0.21	-7.27	0.31	25.8	0.89	10.6	0.23	-5.81	0.35	24.8			10	40	18	21	37	52	8	19	7.4	6.2
	58	0.26	-7.3	0.36	3.8	1.1	1.7	0.24	-4.51	0.41	9.77	1.3	4.65	0.32	-5.89	0.6	4.12			5	37	24	30	50	94	10	8	7.5	8.6
	56	0.22	-5.27	0.35	9.46	1.2	4.83	0.25	-1.19	0.3	0.7	1.3	3.01	0.35	-3.18	0.53	1.85			31	40	28	30	39	60	10	19	0.3	0
NFM	78	0.24	-6.14	0.32	4.21	1.4	7.18	0.23	-7.77	0.32	11.4	1.7	8.35	0.29	-3.63	0.47	4.33			16	14	3	3	28	11	3	15	13.6	3.8
	33	0.18	-8.35	0.3	17.7	1.5	3.45	0.18	-13	0.37	14.3	1.3	4.31	0.24	-2.16	0.39	15.1	1.4	1.84	11	28	24	28	42	74	8	19	13.2	10.5
	49	0.24	-4.96	0.34	10.4	1	0.06	0.28	-2.75	0.35	13	0.94	8.91	0.31	-6.29	0.5	10.1	1.4	2.08	15	36	20	17	38	63	10	17	12.9	6.3
	40	0.21	-3.9	0.32	11.5	1.2	3.27	0.16	-2.69	0.29	5.19	1.4	3.88	0.24	1.8	0.37	10.7	1.4	3.86	4	36	12	16	46	51	10	11	13.3	13.6
	20	0.21	-29.8	0.36	13	1.1	4.74	0.21	-17.8	0.3	11.5	0.75	6.46	0.22	-17.2	0.42	4.06	1.3	2.99	1	30	17	15	45	65	9	15	15.1	10.8
	43	0.2	-5.72	0.34	11.5	1.2	2.3	0.2	-2.99	0.32	13.6			0.24	-2.67	0.39	11.6	1.8	1.36	3	27	15	15	41	76	10	19	14.1	11.8
	40	0.19	-10.7	0.27	17.5			0.22	-9.25	0.35	15.9	1.2	5.78	0.26	-7.8	0.38	14.5	1.3	4.24	5	38	18	25	45	66	10	13	17.1	8.2
	43	0.21	-9.38	0.29	26.4	1.2	4.44	0.34	-1.15	0.4	1.46			0.34	-4.42	0.49	-0.89	1.2	3.58	1	30	25	25	42	64	9	10	13.3	8.4
	43	0.27	-4.7	0.4	8.07	1.3	2.53													22	34	24	28	48	83	10	17	12.8	9.6
	56	0.2	-6.19	0.34	11.5	0.98	4.51	0.19	-2.88	0.38	14.5	1	3.53	0.24	-0.71	0.37	11.2	1.3	4.16	4	38	18	25	45	66	10	14	13.4	7.7
	47	0.19	-31.2	0.28	18.9	1.4	4.79	0.2	-16.8	0.3	7.94			0.23	-20.9	0.32	16	1.3	5.55	16	28	21	21	30	52	7	19	13.9	7.8
	78	0.26	0.26	0.39	12.8			0.38	-2.64	0.47	23.8			0.26	-0.64	0.33	4.97			5	36	20	22	31	60	9	14	13.4	6.9
	42	0.19	-9.91	0.28	23.4	1.7	1.85	0.19	-4.09	0.3	22.8	0.97	3.57	0.23	-7.18	0.36	14.7	1.4	5.6	1	33	22	20	38	54	7	17	18.9	10.3

Table S3 : neurophysiological and clinical features in AFM and NFM patients. BPI Brain Pain Inventory. MAF; Multidimensional Assessment of Fatigue; NRS: Numerical Rating Scale; WPI Wide Pain Index IENFD Intraepidermal Nerve Fober Density
